# Perioperative capacity and contextual challenges in teaching hospitals of southern Ethiopia: explanatory sequential mixed-methods research

**DOI:** 10.1186/s13741-024-00423-6

**Published:** 2024-06-22

**Authors:** Hailemariam Mulugeta, Abebayehu Zemedkun, Getachew Mergia, Semagn M. Abate, Mintesnot Gebremariam, Bedru Jemal, Getachew Nenko, Genet Gebremichael, Aschalew Besha, Mekonnen B. Aregu

**Affiliations:** 1 Department of Anesthesiology, College of Medicine and Health Sciences, Dilla University, Dilla, Ethiopia; 2 Department of Obstetrics and Gynecology, College of Medicine and Health Sciences, Dilla University, Dilla, Ethiopia; 3 Department of Surgery, College of Medicine and Health Sciences, Dilla University, Dilla, Ethiopia; 4 Department of Healthcare Leadership and Management, School of Public Health, College of Medicine and Health Sciences, Dilla University, Dilla, Ethiopia; 5 Department of Nursing, College of Medicine and Health Sciences, Dilla University, Dilla, Ethiopia; 6 Department of Anesthesia and Critical Care, School of Medicine, College of Medicine and Health Sciences, Hawassa University, Hawassa, Ethiopia; 7 Department of Environmental Health, School of Public Health, College of Medicine and Health Sciences, Dilla University, Dilla, Ethiopia

**Keywords:** Surgical capacity, Perioperative capacity, Surgical care, Safe surgery, Patient Safety, Universal health coverage, Global surgery, Explanatory sequential mixed-methods research, Ethiopia

## Abstract

**Background:**

Previous Ethiopian literature on surgical capacity and challenges has focused on quantitative investigations, lacking contextual understanding. This explanatory sequential mixed-methods research (MMR) aimed to assess perioperative capacity and contextual challenges at three teaching hospitals in southern Ethiopia.

**Methods:**

A quantitative survey assessed workforce, infrastructure, service delivery, financing, and information systems. The survey findings were explained by qualitative semi-structured interviews of twenty perioperative providers. Descriptive statistics were integrated with qualitative thematic analysis findings using the narrative waving approach. Key findings from both datasets were linked using a joint display table.

**Results:**

The survey revealed shortages in the specialist workforce (with a ratio of 0.58 per 100,000 population), surgical volume (at 115 surgeries per 100,000 population), equipment, supplies, financing, and perioperative data tracking. Hospitals’ radiology services and blood products were only available 25–50% of the time, while anesthetic agents and essential laboratory services were often available 51–75% of the time. Perioperative management protocols were used rarely (1–25% of the time). Over 90% of patients lack health insurance coverage. Qualitative data also revealed scarcity of perioperative resources and equipment; unaffordable perioperative costs, lack of health insurance coverage, and unforeseen expenses; poor patient safety culture and communication barriers across the perioperative continuum of care; workforce shortages, job dissatisfaction, and concerns of competence; and weak national governance, and sociopolitical turmoil, and global market volatility exacerbating local challenges. These challenges are linked to risks in quality of care and patient safety, according to clinicians.

**Conclusion:**

The study identifies deficiencies in the health system and sociopolitical landscape affecting safe surgery conduct. It highlights the need for comprehensive health system strengthening to expand workforce, upgrade facilities, improve safety culture, resilience, and leadership to ensure timely access to essential surgery. Exploring external factors, such as the impact of national governance and sociopolitical stability on reform efforts is also essential.

**Supplementary Information:**

The online version contains supplementary material available at 10.1186/s13741-024-00423-6.

## Introduction

About five billion people lack safe, timely, and affordable perioperative care, with 94% in low or middle-income countries (LMICs), compared to 14.9% of the population in high-income countries (Meara et al. 2015). The problem can be related to preparedness and policy, service delivery, and the financial burden of surgical care. Accordingly, significant delays in accessing surgical care, compounded by a malfunctioning referral system,critical workforce shortages; inadequate infrastructure; supply chain difficulties; catastrophic expenditures and medial impoverishments; and a lack of surgery-specific policies and priorities, have been reported (Meara et al. 2015), (Scott et al. 2017), (Albutt et al. 2018).

The Ministry of Health of Ethiopia launched the “saving lives through safe surgery (SaLTS)” initiative to improve access to safe surgical and anesthesia care. The project, spanning 2016–2020, aimed to improve access to essential and emergency care. However, evaluations showed underutilization of checklists, limited access to services, and poorly staffed facilities. Most patients were forced to pay out-of-pocket for surgical care, as hospitals struggle to finance perioperative care (Meshesha et al. 2022), (Merga et al. 2023).

Previous literature on Ethiopian perioperative capacity and challenges has focused on quantitative investigations (Bashford 2014), (Burssa et al. 2017), (Kelly et al. 2018), (Kifle et al. 2021), which do not fully incorporate the voices of healthcare professionals (HCP) and patients. This lack of contextual understanding of perioperative care challenges highlights the need for more comprehensive research to address the unmet needs of perioperative care in Ethiopia.

The study aimed to understand how qualitative data on HCP’s perspectives on contextual challenges in perioperative care can explain quantitative results about perioperative capacity measured on a hospital survey. It assessed current surgical capacity in terms of workforce, infrastructure, service delivery, financing, and information management and explored how HCP’s perspectives provide a more comprehensive understanding of these challenges. The study used explanatory sequential MMR to produce current perioperative evidence by integrating a quantitative hospital walkthrough survey with follow-up semi-structured interviews.

## Methods

### Study setting, context, and participants

Ethiopia, the second most populous country in Africa, has a population of over 123 million according to the Population Reference Bureau Population Reference Bureau (Population Reference Bureau (PRB) 2022). Hospitals are divided into primary, general, or specialized, based on the hierarchy of specialization. Public teaching hospitals are semiautonomous and managed by universities with federal government support.

This study is part of an ongoing project aimed to investigate the quality of perioperative care provision in three subthemes: current perioperative capacity and challenges, patient-reported measures, and perioperative outcomes from public teaching hospitals in two regions of southern Ethiopia: Southern Nations, Nationalities, and People Region and Sidma regions. Here, we present the results of the first subtheme, conducted between September 1, 2022, and January 30, 2023, in three teaching hospitals (Dilla University General Hospital (DUGH), Hawasa University Specialized Hospital (HUSH), and Welayita University Specialized Hospital (WUSH)). Interviews were conducted with perioperative clinicians (surgeons, anesthetists, and nurses), who were structurally sampled based on their professional designations.

### Study design, approach, and worldview

**Fig. 1:**
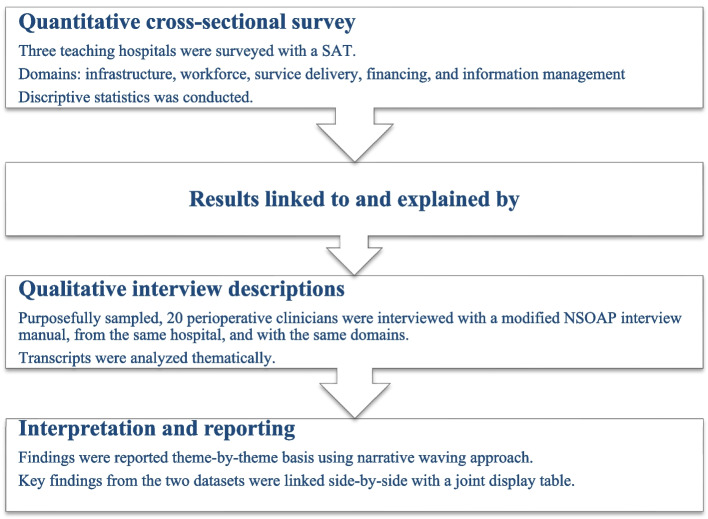
An explanatory sequential mixed-methods research procedure and chain of evidence diagram. Abbreviations: SAT, Surgical Assessment Tool; NSOAP, National Surgical, Obstetric, and Anesthesia Planning

The study utilized a pragmatic worldview and an explanatory sequential MMR design, collecting and analyzing quantitative and qualitative data (Creswell and Clark 2018). The cross-sectional quantitative study established linkages and aimed to address the study’s purpose, while the descriptive qualitative study explained quantitative results and provided context. Methodological integration involved surveying surgical capacity in hospitals, followed by interviews with perioperative clinicians. The findings were reported in accordance with the American Psychological Association’s Mixed Methods Article Reporting Standards (Levitt et al. 2018). Figure 1 illustrates the chain of evidence and an overview of the design of the study.

### Sample size and sampling technique

Three teaching hospitals in southern Ethiopia were selected for a convenient sampling technique. In-depth interviews were conducted with perioperative clinicians, with 16 interviews being the minimally workable number at which one may expect to achieve theoretical saturation (Vasileiou et al. 2018). Twenty interviews were conducted with seven surgeons, six anesthetists, and seven nurses.

### Variables

Attributes of perioperative capacity, such as percent availably of equipment and drugs, supplies, surgical volume-to-population ratio, and specialist-to-population ratio, along with the factors determining the perioperative capacity, were assessed in five domains: namely, workforce, infrastructure, service delivery, financing, and information management.

### Data collection instruments, and procedures

#### Quantitative phase

The Surgical Assessment Tool (SAT) was used in a hospital walkthrough survey developed by the Harvard Program in Global Surgery and Social Change (PGSSC) and the World Health Organization (WHO) to assess factors determining perioperative capacity World Health Organization (World Health Organization (WHO) 2017). The survey categorized the availability of general hospital infrastructure, radiology and laboratory services, drugs, blood supply, quality and safety assurance, operative room equipment and supplies, and prospective patient outcome tracking mechanisms on a 6-point Likert scale: 0 (never) to 100% (always). The information was collected through direct observation during hospital inspections, in-person interviews with clinical coordinators, and reviews of perioperative documentation or the Health Management Information System (HMIS).

#### Qualitative phase

The study utilized the National Surgical, Obstetric, and Anesthesia Planning (NSOAP) interview manual, a collaborative initiative between United Nations Institute for Training and Research, PGSSC, and Global Surgery Foundation United Nations Institute for Training and Research (United Nations Institute for Training and Research (UNITAR): National Surgical Obstetric Anesthesia Planning (NSOAP) Manual. 2023). The tool contains semi-structured interview questions on infrastructure, workforce, service delivery, financing, and information management. To gather data in Amharic, key parts of the NSOAP tool were translated for surgeons, anesthetists, and nurses. Our team then conducted internal testing to ensure the translations were accurate, culturally appropriate, and maintained the clarity and comprehensibility of the interview guide. Purposive sampling was used, with senior-level perioperative clinicians being interviewed. Consent was obtained before enrollment, and interviews were conducted in private. The Amharic recordings were transcribed, cross-checked for accuracy, and translated to English.

The study employed various strategies to verify the validity of qualitative data. These included using an audio recorder to confirm the accuracy of interpretations. Dependability was maintained through recording participants’ interviews, taking notes, and transcribing verbatim. A code-recode method and independent coder analyzed the data. Transferability was achieved by collecting interview data from purposively selected perioperative healthcare professionals. Conformability or neutrality was confirmed by using participants’ words from interview transcripts to confirm that the data interpretation accurately reflects participant words.

### Data analysis, interpretation, and reporting

#### Quantitative phase

The survey data was analyzed using SPSS 25.0 (Chicago, IL, USA), with mean (SD), numbers, and percentages presented. The mean availability of perioperative infrastructure, including drugs and equipment, was analyzed by transforming related Likert scale variables into new variables. For example, availability of ketamine, propofol, and thiopental were transformed into “availability of intravenous anesthetics.”

#### Qualitative phase

A codebook was developed by authors HM and AZ, covering five themes: infrastructure, workforce, service delivery, financing, and information management. Thematic analysis was used by GN and AZ to code transcripts, with independent validation by a third analyst (MBA). MAXQDA 24 (Berlin: VERBI software, 2023) software was used for coding.

#### Integration of findings

To integrate quantitative and qualitative data at the interpretation and reporting level, two approaches were used: integrating through narrative and through joint display. Initially, the two datasets were reported using a narrative waving approach, in which quantitative and qualitative findings are combined “theme-by-theme” or “concept-by-concept.” Then, a joint display table is used to summarize the key findings and to establish a linking activity between the two datasets. This linking activity searches for related quantitative findings and chooses appropriate qualitative quotes to illustrate them. Colored arrows are used to indicate convergence, complementarity, expansion, or divergence between the findings (Fetters et al. 2013).

### Assumptions and reflexivity

Our research team included both academic researchers with public health expertise (GN and MBA) and medical professionals who work in perioperatively (anesthetists: HM, AZ, SMA, BJ, and AB; surgeons: GM and MG; and a specialized nurse: GG). In conducting the interviews, we tried to accurately capture participants’ own stories about their perioperative experiences and perspectives. We recognized that our own experiences and assumptions might influence how we interpret what participants say. To address this, our research team discussed any potential biases, personal views, and how our understanding of the research questions might change over time. We considered these factors when analyzing the interviews and interpreting the results. We also acknowledge that participants’ experiences and social interactions shape their understanding of perioperative care. Therefore, we viewed their narratives as extending beyond individual experiences, offering a broader perspective on how healthcare professionals navigate the intricate social dynamics of the surgical environment.

### Declaration of generative AI and AI-assisted technologies in the writing process

During the preparation of this work, the authors used the Paperpal word plugin to improve language and readability. After using this tool, the authors reviewed and edited the content as needed and take full responsibility for the content of the publication.

## Results

The MMR on perioperative care challenges involved a quantitative survey and semi-structured interviews with 20 perioperative clinicians. The data were presented in five domains: infrastructure, service delivery, workforce, information management, and financing. Each domain’s qualitative findings were connected to the quantitative survey findings, and three additional subthemes emerged from the inductive analysis of interview data: sociopolitical landscape, leadership and governance, and global events (such as COVID-19 and market volatility).

### Infrastructure

The three teaching hospitals, DUGH, HUSH, and WUSH, serve about 23 million people. About 26,272 surgical admissions (approximately 115 surgeries per 100,000 population) were recorded over 1 year (January 01 to December 25, 2022). Obstetric and gynecologic surgical procedures comprised approximately 74.58% (19,593/26,272) of the total surgical admissions, while the rest were from other surgical departments, including orthopedics. The hospitals’ infrastructure was not readily available during the hospital walkthrough survey. Table 1 displays the mean frequency of the availability of perioperative infrastructure over the 5-month study period.

**Table 1:** Mean availability of perioperative infrastructure in three teaching hospitals of southern Ethiopia, Sept. 1, 2022, to Jan 30, 2023

	**Mean (SD)**	Frequency of availability*
General infrastructure (running water, regular 24/7 electricity, internet connectivity, and oxygen source)	4.07 (0.42)	Often (51–75%)
Essential perioperative medications (anesthetics and emergency drugs)	4.03 (0.25)	Often (51–75%)
Blood products	2.67 (0.58)	Sometimes (26–50%)
Radiology (X-ray, CT-scan, and ultrasound) service	3.42 (1.38)	Sometimes (26–50%)
Essential laboratory tests (CBC, chemistry panel, coagulation profile, infectious panel screen, liver function test, urinalysis)	3.50 (1.00)	Often (51–75%)
Adult size anesthetic equipment	4.67 (0.17)	Almost always (76–99%)
Pediatric size anesthetic equipment (ETT, LMA, laryngoscope, Magill forceps, oropharyngeal airway, BVM)	3.47 (0.50)	Sometimes (26–50%)
General operation room equipment and supplies (antiseptics, autoclave, electrocautery, chest tube, forceps, stiches, etc.)	3.94 (0.22)	Often (51–75%)

^*^The mean values were approximated to the nearest whole number without considering standard deviations to determine frequency of infrastructure availability. Values: 1 = never (0%), 2 = rarely (1–25%), 3 = sometimes (26–50%), 4 = often (51–75%), 5 = almost always (76–99%), and 6 = always (100%)

*Abbreviations:* *BVM* bag-valve-mask, *CBC* complete blood count, *CT* computed tomography, *ETT* endotracheal tube, *LMA* laryngeal mask airway, *SD* standard deviation

Two themes were generated from the interview data: scarcity and system breakdown (inadequate equipment and supplies, and malfunctioning infrastructure) and cascading impact on frontline workflow, with shortages of anesthetic drugs and inadequate laboratory and radiological services being the most recurring concerns. Clinicians reported these issues hindering patient care, causing surgery cancelations, delays, errors, and an increased risk of complications. A participant commented,

*“…when it comes to materials and equipment, for example, cautery machines, autoclaves, and suction machines are not enough or some of them are not functioning well. … there has been cancellation of surgical cases due to shortage of anesthetic medications.”* (Participant 1).

Talking about perioperative efficiency and workflow, a clinician said:

*“The workflow in the operating room is inefficient, and there is a lack of space and the number of OR tables are not adequate.”* (Participant 2).

The MMR found that blood shortages are a significant challenge in hospitals, with availability only 26–50% of the time. The mean time to get in-stock and out-of-stock blood after placing an order is 0.42 (0.14) and 13.67 (9.61) h, respectively. Perioperative clinicians acknowledged that these shortages resulted surgery cancelations, treatment delays, and even death. Factors contributing to these shortages include declining blood donations, increased demand, and issues with blood collection and storage. The excerpt below highlights the many challenges that hospitals face when blood supply is short.

*“Another major barrier to the cancellation of the cases is blood shortages…, and it is usually difficult to get type-specific blood; and there are more cases of blood shortage related cancellations than for other reasons; and there is also a problem with blood collection in blood banks and a policy problem at a national level. In the past for example, hospitals used to collect from volunteering patient attendants or family—it was a practical alternative to replacing hands-on blood.”* (Participant 3).

Participants’ account revealed that problems span from poor blood collection practices in blood banks to the national policy issues hindering effective blood management. It suggests that a multifaceted approach is needed to address blood collection practices, storage policies, and potential policy changes to ensure a reliable blood supply for surgical procedures.

Overall, the study revealed perioperative challenges due to a shortage of essential equipment and supplies, including anesthetic drugs, blood products, laboratory services, and operating room supplies, with a cascading effect to frontline care delivery.

### Service delivery: surgical volume, quality, and safety

Of the total surgical admissions over a year, Bellwether procedures comprised about 41.38% (10,872/26,272). Cesarean deliveries contributed the highest (9027/10,872, 83.03%) among the Bellwether procedures followed by open fracture repair including fixation (1049/10,872, 9.65%) and laparotomy (796/10,872, 7.32%). Further analysis of the survey data revealed that the WHO surgical safety checklist and perioperative monitoring standards were used 76–100% of the time, whereas there was poor compliance to the use of perioperative protocols or guidelines (1–25% usage rate). Table 2 shows the summary statistics for mean frequency of usage of quality and safety assurance mechanisms.

**Table 2:** Mean usage rate of quality and safety assurance mechanisms in three teaching hospitals of southern Ethiopia, Sept. 1, 2022, to Jan 30, 2023

	**Mean (SD)**	Frequency of use *
Use of WHO surgical safety checklist	4.67 (0.58)	Almost always (76–99%)
Use of pulse oximetry monitoring	6.00 (0.00)	Always (100%)
Use of non-invasive blood pressure monitoring	5.00 (0.00)	Almost always (76–99%)
Use of other standards of monitoring (ECG, capnograph, and Temperature)	4.67 (0.58)	Almost always (76–99%)
Use of management guidelines/protocols during emergency care	2.00 (1.00)	Rarely (1–25%)
Use of management guidelines/protocols during surgery	2.00 (1.00)	Rarely (1–25%)
Use of management guidelines/protocols (e.g., checklists for routine machine check, pre-anesthesia preparation, adverse event) during anesthesia care	4.33 (0.58)	Often (51–75%)

^*^The mean values were approximated to the nearest whole number without considering standard deviations to determine frequency of use of quality and safety assurance mechanisms. Values: 1 = never (0%), 2 = rarely (1–25%), 3 = sometimes (26–50%), 4 = often (51–75%), 5 = almost always (76–99%), and 6 = always (100%)

*Abbreviations:* *ECG* electrocardiograph, *SD* standard deviation

Three themes were constructed from the qualitative interviews of clinicians related to service delivery: poor patient safety culture (PSC) such as poor compliance to and incomplete use of safety checklists, protocols, and guidelines, top-down approach quality improvement (QI), and fragmented perioperative communication.

Most interviewees stated that there is incomplete documentation of perioperative care and they do not always use the WHO safety checklist. They also noted the lack of existing perioperative PSC and poor compliance to the utilization of protocols and guidelines. For example, a clinician expressed the issue as follows:

*“… there is a WHO checklist and that is good at least we have started it, but it is only filled the ‘time out’ section most of the time; I mean, we don’t fill in a ‘sign in’ and ‘sign out’—and it often differs from one professional to another; and sometimes they forget attaching it with a patient chart.”* (Participant 5).

Another said:

*“There have been many instances of implementation training and team meetings to improve our documentation problems and to fill existing gaps, but there is no tangible change.”* (Participant 15).

Furthermore, clinicians believe that there is inadequate interprofessional communication, collaboration, and team-based care among hospital departments, leading to delays in care, errors, and patient harm. For example, a clinician said:

“*We sometimes take part in quality meetings but not often.”* (Participant 13).

Another described it as follows: *“The surgeon may not be aware of the patient’s medical history, or the nurse may not be aware of the surgeon’s instructions.”* (Participant 12).

The interviews also revealed that perioperative QI initiatives are not sustainable and challenging to improve care over time. For instance, one interviewee explained the issue as

*“there are some quality improvement projects, like SaLTS* [saving lives through safe surgery]*, that are conducted to increase OR efficiency and quality of surgical and anesthesia care. The problem, however, is that projects often begin, but we do not see a sustainable change. The baseline problem usually recurs in the middle of the projects or after completion.”* (Participant 8).

Taken together, the qualitative themes focused on the pervasive weaknesses in the perioperative safety culture and the ineffectiveness of current improvement efforts. The excerpts suggest a sense of frustration and powerlessness among the clinicians, with a pessimistic overall tone regarding the current state of perioperative practices. While they acknowledge their participation in some practices like safety checklists, they also express a lack of tangible change despite repeated training and meetings. This suggests that they may not feel fully empowered to implement or enforce stricter protocols. The lack of sustainable improvement suggests a need for a more comprehensive approach to addressing the root causes of the quality and safety issues.

### Surgical workforce

Combining all the surgical, obstetric, and anesthesia (SOA) specialist workforce together, there were only 0.58 practitioners per 100,000 population at the participating teaching hospitals. There were 62 OR nursing practitioners (mean (SD), 13.3 (4.9) per hospital). Table 3 illustrates the surgical workforce volume at the surveyed hospitals during data collection period.

**Table 3:** Perioperative workforce density in three teaching hospitals of southern Ethiopia, Sept. 1, 2022, to Jan 30, 2023

	**Workforce number**	**Mean (SD)**	**Workforce per 100,000 population**
Nationally certified physician surgical specialists *	45	15.0 (6.6)	0.20
Nationally certified physician obstetric specialists	26	9.3 (2.5)	0.10
Nationally certified physician anesthesia specialists	6	2.0 (1.7)	0.03
Nationally certified nonphysician anesthesia specialists #	57	19.0 (5.6)	0.30
Nationally certified nonphysician anesthesia professionals	40	13.3 (4.9)	NA
Operating room general nursing practitioners	62	13.3 (4.9)	NA

*Abbreviations:* *NA* not applicable, *SD* standard deviation

^*^Surgical specialists in this study include general surgeons, orthopedic surgeons, and other subspecialists, such as thoracic and pediatric surgeons

^#^In Ethiopian context, a nationally certified nonphysician anesthesia specialists are those practitioners who completed 4 + years of bachelor’s and 2 years of master’s degree in clinical anesthesia

The interview data analysis produces two major themes related to the workforce, which are job dissatisfaction and uncertainties about workforce volume. Concerns regarding lack of job satisfaction were more widespread among healthcare professionals. They raised several factors, including unfair pay, lack of recognition, lack of health insurance, high workload, and poor workplace conditions. For example, one clinician said,

“*I don’t think the country’s salary for health professionals goes with standard of living, especially from the current inflation.”* (Participant 6).

Perioperative clinicians’ workforce volume is a topic of debate, with some arguing a shortage, others criticize the quality of health professional education and subspeciality care. For example, a participant put it as follows:

“*There is still a shortage of human power per the capacity this hospital can deliver. If I tell you the OR nursing standard for example, there should be minimum of three nurses per table, I mean scrub, circulator, and runner; but it has not been possible until now in this hospital.”* (Participant 1).

Another said:

“*…the number of professionals is increasing now; on top of that, the shortage of human power has decreased because of the increase in residents [Surgical fellow students] intake capacity. … We cannot say that there is a shortage of human power per the capacity this hospital can deliver. However, there are still no subspecialist surgeons; for example, there are many patients who are referred to another institution for neuro, thoracic and pediatric surgery.”* (Participant 9).

In general, the findings revealed that hospitals were understaffed, with the SOA specialist workforce below 1 per 100,000 population per cadre. Also, providers reported that there is job dissatisfaction among clinicians which might have an indirect effect on the quality of perioperative care they deliver.

### Budget and financing

A survey of participating hospitals revealed that less than 10% of patients had health insurance coverage for perioperative care. The out-of-pocket costs of surgical care were unknown. However, the hospitals covered the cost of maternity care, including cesarean delivery, for free. Analysis of interview data showed that costly perioperative care, insufficient or nonexistent health insurance, insufficient hospital funding for surgical care, and treating patients who lacked insurance or financial resources raised ethical and moral concerns among clinicians.

Providers recognize that financial barriers to surgical care impact patient care-seeking behavior, leading to delays. They occasionally offer free care, particularly for emergency cases. For example, one clinician said,

“*Most of the patients come to government medical facilities with the assumption of getting medical care with minimal payment or for free; and many say, ‘we don’t have the money’ after the procedure is done—it is the biggest challenge.”* (Participant 5).

Another said,

*“Sometimes a patient who can’t pay comes for surgery, especially for emergency surgery, and we use OR reserve supplies, to provide the service—you can’t deny an emergency care; and sometimes we, professionals, even spend money ourselves and buy important supplies for patients; but there’s no other way to provide care for such patients.*” (Participant 20).

Providers also reported that government health insurance does not cover all surgical procedures, and many patients lack coverage. Commenting on this issue, one interviewee said,

*“There are a small number of patients with health insurance; what’s the problem? most of the supplies are not found in hospitals, including anesthetic agents; it means that patients buying from a private pharmacy will be covering the expense by their-selves, and you know it is unintended.”* (Participant 18).

Moreover, clinicians argue that the government lacks sufficient funding for surgical care, resulting in shortages of essential supplies and equipment. The availability of anesthetic drugs and other surgical supplies is often limited. They suggest a need for upscale surgical care financing. Explaining this matter, one clinician said,

*“The biggest problem is that availability of the hospital’s supply of anesthetic drugs and other surgical supplies, such as gloves, catheters, stiches, and so on, and it runs out quickly and often. There is a need to upscale surgical care financing. I don’t believe that the authorities well consider the surgical service.”* (Participant 8).

A lack of clarity exists regarding providing care to patients who cannot afford or lack insurance. Providers acknowledge delays in care, cancelations of procedures, and complications due to delayed care. A participant put it as follows:

*“The worst thing is when a patient who can’t pay comes to us. It is extremely hard, especially in emergency situations, because every minute and hour count on the patient’s outcome. It’s clear that the outcome will be poor as such patients wait more time to get the service, but we can’t do anything as professionals—we have no option.”* (Participant 14).

Clinicians often face ethical and moral dilemmas when patients cannot afford surgical care. They must decide whether to provide care to those who cannot pay or turn the patient away. For example, a provider stated,

*“sometimes you may get leftover OR supplies from other patients; so, we, the surgical team, let emergency patients get the surgical services for free, but it is not always possible. It is a moral burden as a clinician to decide on such issues. I believe hospitals should have a mechanism for such patients, like a social service or something else.”* (Participant 19).

Overall, the MMR reveals inadequate hospital financing and high perioperative cost. Qualitative themes focused on the ethical and moral burdens and frustration faced by clinicians due to the excessive cost of perioperative care, insufficient financial resources, and lack of a clear policy on providing care to those who cannot afford it. While clinicians acknowledge some resourcefulness, like using leftover supplies, they primarily view themselves as bystanders in a situation beyond their control. The overall tone is pessimistic as clinicians are caught between providing necessary care and the limitations imposed by the financial system. They emphasize the need for improved financing and clear guidelines to address these ethical dilemmas.

### Information management

Two out of the three surveyed teaching hospitals had electronic HMIS at admission, but all the perioperative data recording was paper based. Further survey analysis revealed that perioperative patient outcome data, such as postoperative mortality and complications, anesthesia-related adverse events, and nursing care adverse events, were collected only sometimes (26–50% of the time). In contrast, two themes emerged from the interview transcript: fragmented and inconsistent health data recording and poor support system for research and quality improvement (QI) projects.

The clinicians reported that the information management system at their hospital is not adequate. This includes problems with accessing patient information, lost records, and a lack of coordination between different departments. For example, an interviewee said,

“*For new patients coming to our hospital, their information is now computerized; but once they are triaged to each ward, they get a paper-based medical card; some medical charts may be lost; and sometimes we have difficulty obtaining a prior medical history.”* (Participant 9).

Another said:

“*Sometimes the card number of patients can be lost; sometimes, if it’s soon enough, some clinicians who have given the medical care may remember the patients’ past situation, and they’re issued a new medical card number. But it is not good practice, I believe.”* (Participant 18).

Interviewees acknowledged that the challenges related to health information management make it difficult to track a patient’s progress and to provide continuity of care or may even have a medicolegal concern. Explaining this issue, a clinician said:

“*There were also times when we met a medicolegal issue, and we lost a patient’s medical record. We need to improve the way we manage patient health data and the hospital information system in general.”* (Participant 11).

The providers also reported that there is difficulty accessing perioperative data for research purposes and that lack of funding for perioperative QI projects. This makes it difficult to track patient outcomes and identify areas for improvement. A clinician commenting on this theme said:

“*the experts who access the patient charts do not easily cooperate, … I’m not sure but I haven’t ever heard a hospital budget that is specifically funded for QI projects or clinical audit purpose; We need a system in place to collect data and track patient outcomes. We also need funding to support research projects.”* (Participant 6).

In summary, this study found that most of the health data recording was paper-based; that there was no a clear mechanism to track perioperative outcome data prospectively over time; that there was poor health data management system, such as poor documentation, difficulty in accessing prior patient information, and a lack of coordination between different department; and that there was poor support system for research and QI projects.

### Sociopolitical landscape, leadership and governance, and global events

The clinicians reported that the sociopolitical unrest in Ethiopia has also had a significant impact on the hospital’s ability to provide safe and quality perioperative care. This includes violence and instability in the region, which has made it difficult to get perioperative supplies due to disruptions to supply chains; a decrease in government funding for healthcare, which has led to cuts in services; and a decrease in public trust in the healthcare system. For example, a provider explained the issue as follows:

*“… the price of medications and other supplies is being increased from time to time; it’s often hard to find the materials we want; it’s hard to say that we’re providing standardized healthcare service; and I think all this problem is related to the social unrest in the country. And you know we have been in political turmoil, even we had a civil war [occurred in northern Ethiopia]. These are big issues for all these shortages of the basic needs in healthcare.”* (Participant 2).

Another said:

*“domestic civil and political unrest and global market instability have a significant impact on our medical delivery; it’s hard to give quality surgical care. The community already has financial hardship, it’s become harder and harder to pay for medical expenses.”* (Participant 12).

While some clinicians acknowledge the disruptive effects of COVID-19 on the global market and healthcare delivery, they generally view it as an exacerbating factor rather than the root cause of problems. They argue that sociopolitical instability creates a more significant challenge, making it difficult to respond effectively to the pandemic. For example, one participant put it as follows:

*“COVID-19 has disrupted the global market; I understand that, but it is affecting all not only us; if we were stable inside, its impact would have been negligible, because you can see many countries, including those in Africa, have already recovered from it. So, our internal unrest takes the lion’s share; and COVID is a complement.”* (Participant 3).

Another said:

“… *COVID has shown us how our healthcare system is poor; … there is an improvement in IPC [infection prevention and control]—our facemask use is improved; for example, there were professionals who entered the OR without wearing a mask. However, much has changed since the coronavirus—the overall healthcare system has been disrupted since then; surgical supplies and medications have been expensive.”* (Participant 10).

Finally, perioperative providers have identified a lack of good leadership and governance at both local and national levels as a significant challenge in the healthcare system. They have reported corrupt and unskilled healthcare leaders and a lack of prioritization among authorities. For example, one clinician expressed the issue as follows:

“*What worries me most is, the authorities do not seem to be ready to change the problems of the healthcare system; some of them are corrupted and others are politically delegated without any healthcare leadership training or lack the necessity experience. Overall, I believe the administrative perspective towards healthcare, particularly for surgery, must be improved, I mean, here at hospital or the university and at national level.”* (Participant 7).

Together, participants’ narratives highlighted the detrimental impact of external factors, such as weak national governance, sociopolitical instability, resource constraints, and global market volatility on the hospital’s ability to deliver safe and quality perioperative care. The overall tone is pessimistic regarding the ability to provide optimal care under these circumstances. Therefore, the findings suggest that clinicians feel impacted by these external factors, rather than being a direct cause of the problems.

**Fig. 2:**
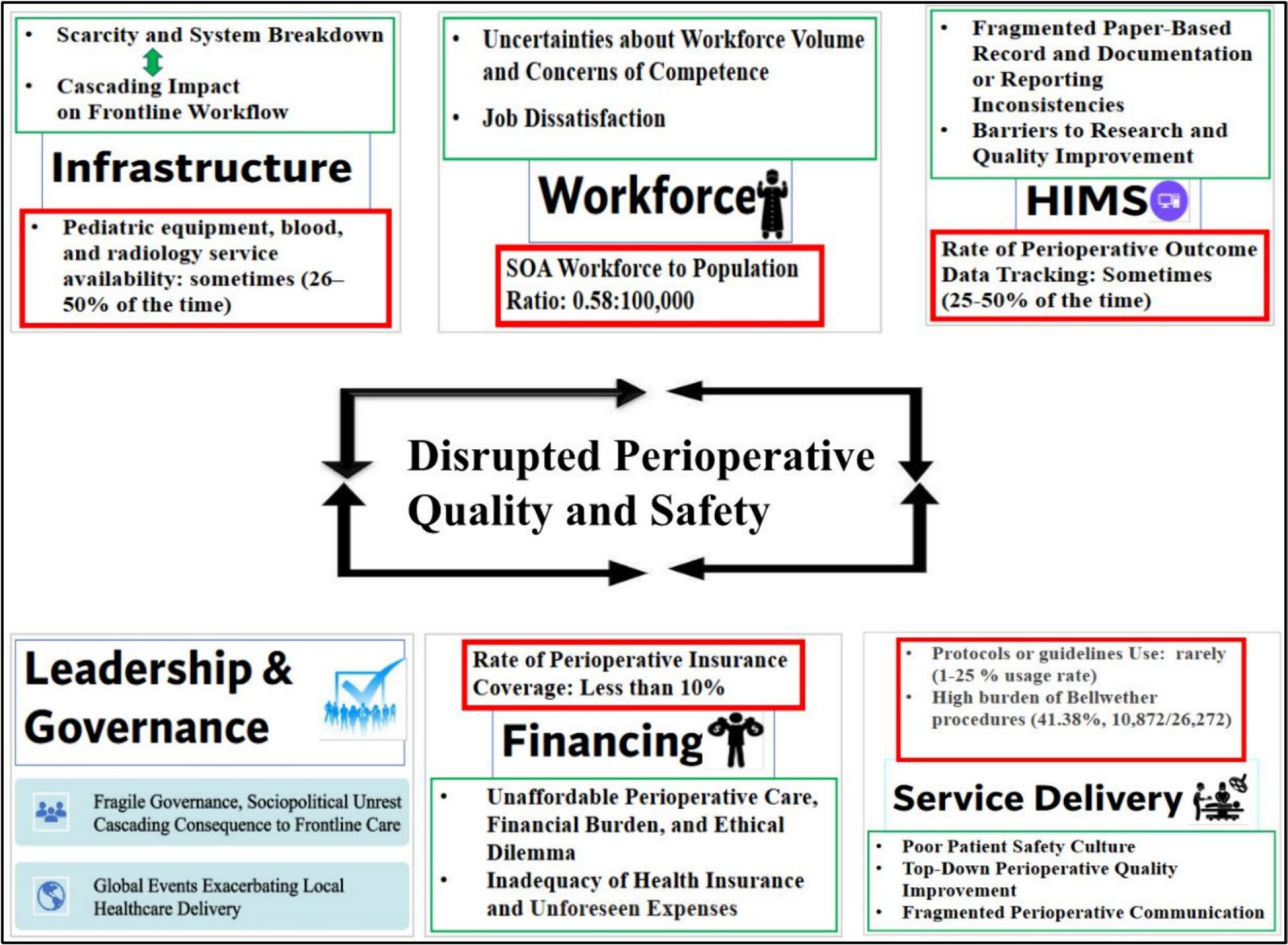
A concept map of interconnected perioperative care challenges and associated quality and safety disruption, 2023. Note: Red text boxes imply illustrative quantitative data points for each domain. Abbreviation: HIMS, health information management system; SOA, surgical, obstetrics, and anesthesia

Putting all findings together, this MMR revealed persisting major gaps in Ethiopian perioperative care. A joint display table linking the key survey findings of perioperative hospital capacity to quotes of contextual challenges perceived by clinicians is shown as an additional.pdf file. In this table, meta-inferences are shown with colored arrows, indicating convergence (agreement), complementarity (different but non-contradictory interpretations), or expansion (allowing for overlap and further interpretation) between the findings. There was no divergence (conflicting interpretations) during the linking activity (Additional file 1). In addition, the schematic concept map in Fig. 2 visually depicts the intricate interplay among the key qualitative themes and survey results. This visualization displays how these interconnected challenges disrupt the quality and safety of perioperative care within the system.

## Discussion

Perioperative performance is a complex process that requires providers to coordinate team-based care that is timely, safe, effective, and patient-centered (Chazapis et al. 2018), (Baker 2001). The study found several contextual challenges that hinder the delivery of quality perioperative care at three teaching hospitals in southern Ethiopia. These challenges include inadequate infrastructure with 115 surgeries per 100,000 people, poor use of safety assurance mechanisms, lack of interprofessional collaboration, and high burden of emergency bellwether procedures accounting about 41% of the total surgical admissions. Additionally, there is a critical shortage of perioperative workforce density, with 0.58 SOA specialists per 100,000 population, compounded by job dissatisfaction due to substandard working conditions, workload, and unfair earnings. Inadequate financing, information management systems, and ongoing sociopolitical unrest, leadership, and governance also hinder the sustainability of national efforts to improve perioperative care capacity and quality. Addressing these challenges is crucial for ensuring timely, safe, effective, and patient-centered care in perioperative settings.

### Perioperative capacity is limited by inadequate infrastructure.

Previous studies have shown that inadequate infrastructure is a significant challenge to perioperative capacity in Ethiopia. A preliminary national survey by Burssa et al. (2017) found that the availability of essential supplies, such as anesthesia machines, ventilators, and surgical instruments, was a major challenge to the implementation of the national government-driven surgical plan, the SaLTS (Burssa et al. 2017). The surgical volume in this study is 115 per 100,000 population, lower than the previous Ethiopian survey finding of 189 by Merga et al. (Merga et al. 2023). These data must be interpreted with caution because their estimate is derived from a national survey that included all levels of surgical facilities. However, both reports show that the challenge persists even after the SaLTS project was implemented over 5 years (2016–2020).

Ethiopia and other sub-Saharan countries consistently fall short of the Lancet Commission on Global Surgery’s (LCoGS) target of 5000 per 100,000 population (Meara et al. 2015). Infrastructure inadequacy is a significant factor, with Liberia reporting 462 surgeries per 100,000 population (Adde et al. 2020). Uganda’s mixed-methods assessment by Albutt et al. (2019) estimated 145 annual operations per 100,000 population (Albutt et al. 2019). Patil et al.’s (2023) systematic review found that in LMICs, an average of 877 surgeries per 100,000 population increased with gross domestic product per capita. India and Brazil reported volumes close to the LCoGS benchmark (Patil et al. 2023). The study suggests that weak healthcare infrastructure, rising medical supply and equipment costs, supply chain disruptions, and health system issues may contribute to low surgical productivity and income levels.

### Poor patient safety culture and high burden of bellwether procedures affect the perioperative service delivery

Inter-professional teamwork, surgical team dialogue, and managerial support significantly impact perioperative care quality and progress. These factors may impact the operating room work environment, including confidence, stress, and job satisfaction (Chazapis et al. 2018; Gelb et al. 2018). The study highlights gaps in perioperative PSC, such as low compliance with checklists and evidence-based protocols. These barriers pose threats to patient safety, particularly in LMICs, and may be related to team awareness, time constraints, and poor organizational PSC (Munthali et al. 2022).

Interviews revealed that poor communication, disjointed handovers, and lack of collaboration negatively impact perioperative care. Patient safety literature emphasizes the importance of teamwork, information sharing, and coordination in perioperative settings (Chazapis et al. 2018; Gelb et al. 2018). Overcoming these gaps is crucial in LMICs like Ethiopia with high surgical disease burdens. Implementation science should be the focus of initiatives to enhance teamwork, communication, shared mental models, and patient safety culture. Effective care in complex perioperative settings requires surgeons, anesthesiologists, and nurses to collaborate well. Learning from top institutions can accelerate cultural transformations needed for effective care (Bonds 2018), (Falcone et al. 2021).

The study highlights the burden of over 80% of all bellwethers performed in obstetric procedures, putting additional strain on perioperative safety. The high emergency-to-elective surgery ratio in LMICs requires significant investment in emergency perioperative care (Prin et al. 2018). To improve patient outcomes, it is therefore crucial to promote a PSC and capacity building, emphasizing safety and teamwork as highly valued as technical skills.

### The perioperative workforce shortage and job dissatisfaction further affect service delivery

The study found that 0.58 specialist SOA per 100,000 population mirrors previous assessments of workforce deficits in Africa. Ethiopia’s 2023 surgical capacity analysis recorded a specialist workforce density of 1.31 providers per 100,000 (Merga et al. 2023), while Liberia’s 2020 study documented 0.33 (Adde et al. 2020). South Africa, an upper middle-income country, has an estimated ten specialists per 100,000 people according to Tiwari et al. (Tiwari et al. 2021), higher than Ethiopia and Liberia’s estimates. These differences may be due to the rise in surgical workforce density as per capita income increases and increased funding for medical education and healthcare. However, (Biccard et al.’s 2018) prospective multinational study across Africa, including South Africa, found a median of 0.7 SOA per 100,000 population (Biccard et al. 2018), indicating that LMICs need to scale up rapidly to meet the LCoGS’s target of 20 SOA specialist providers per 100,000 by 2030 (Meara et al. 2015).

Job dissatisfaction among healthcare workers in Ethiopia is low, with a pooled prevalence of around 46% (Girma et al. 2021). Healthcare professionals have identified insufficient pay, high workloads, limited professional development, lack of recognition, poor relationships with staff and managers, and poor infrastructure as sources of dissatisfaction. Workforce shortages and unfavorable working conditions further exacerbate burnout and intentions to quit (Lrago et al. 2018; Woldekiros et al. 2022). The unfavorable circumstances discourage new trainees from joining or continuing in practice. A 58% turnover intention among Ethiopian healthcare workers is a significant concern (Gebrekidan et al. 2023). To build a strong surgical workforce, increased income, workplace improvements, supervision, recognition, and professional progress are implicated.

### Inadequate perioperative financing and public health insurance could have resulted in catastrophic expenditure and impoverishment

The study revealed that over 90% of patients lack health insurance coverage for surgery and anesthesia, with out-of-pocket payment burdens exacerbated by poor socioeconomic status. Inadequate government funding leads to shortages in supplies, medications, and blood products, forcing clinicians to ration resources or pay out-of-pocket for patients in need. These gaps highlight health financing deficiencies and the need for stronger surgical financing policies to prevent medical poverty and ensure access to essential services like surgery.

High surgical costs and a lack of financial risk protection drive CE and impoverishment in low-middle-income countries (LMICs), particularly in sub-Saharan Africa and Southeast Asia (Meara et al. 2015). Over time, the proportion of medically impoverished individuals has increased globally and within individual countries (Njagi et al. 2018), (Ifeanyichi et al. 2021).

Investing in perioperative care is affordable and promotes economic growth if political will, global cooperation, system-based approach, evidence-based policymaking, and implementation guidance are in place (Meara et al. 2015), (Roa et al. 2019). This study highlights the need for universal financial risk protection policies and adequate domestic financing reforms which are foundational for access to safe and affordable perioperative care. Without progress, surgical care access will remain bifurcated along economic lines.

### Comprehensive perioperative digital health information systems strengthening, research, and data utilization is needed

The study revealed significant deficiencies in perioperative information systems, including paper-based data management, poor medical record access, no systematic tracking of surgical outcomes, and insufficient funding for research and quality improvement projects. The inability to collect and utilize data hinders facilities’ ability to identify safety risks, benchmark indicators, and measure improvement. A robust health information system supporting the creation of a perioperative data warehouse is vital for monitoring services, adverse event detection, feedback, resource allocation, and decision-making. This highlights the importance of investing in integrated digital health information systems and data science capacity in LMICs to strengthen the ability to capture, analyze, and apply perioperative data, enabling facilities to identify care improvements, enhance quality, and promote safety culture (Mughal et al. 2023), (Hofer et al. 2016), (Hung et al. 2020), (Mrara and Oladimeji 2023).

### Issues related with leadership, sociopolitical turmoil, and global events would have impacted perioperative capacity

Ethiopia has faced political reforms, conflicts, and violence (Jima 2021), potentially impacting perioperative care delivery. Interviewees emphasized how civil unrest and instability have disrupted supply chains, decreased healthcare funding, and eroded public trust. Inadequate management at health facilities has led to poor coordination, corruption, and lack of support for quality improvement efforts. Facility leadership plays a crucial role in fostering teamwork, safety protocols, and a culture of learning (Swanwick and McKimm 2011), (Diggele et al. 2020).

Interviewees also emphasize that global events, such as COVID-19 and market volatility, have exacerbated challenges in perioperative capacity, confirming the disproportionate impact of the pandemic and fragmented governance in LMICs. There is a growing push for strengthening and reforming the framework for pandemic preparedness and building global health security capacity (Lal et al. 2022), (Greenhill and Khalil 2023), (Doble et al. 2023).

Overall, the study highlights the extensive effects of leadership, governance, and sociopolitical issues on perioperative safety. Interventions to increase surgical capacity in LMICs like Ethiopia must focus on fostering proactive and transparent governance systems at the national and individual health facility levels.

### Limitations

This mixed-methods study advances understanding of perioperative care challenges by incorporating healthcare providers’ voices. The study’s mixed-methods design offers richer insights into quantitative perioperative capacity assessment findings. However, it has several limitations. First. it is not possible to generalize to other teaching hospitals in Ethiopia pertaining to the use of convenient sampling. Second, we acknowledge a potential observer, response, or recall biases from the SAT. Third, the study lacks sufficient findings on perioperative mortality and morbidity to support its conclusions. Fourth, we acknowledge that the current study primarily reflects the viewpoints of clinicians directly involved in perioperative care. Financial constraints limited the inclusion of hospital administrators and other perioperative staff, whose insights could have provided a more comprehensive picture. Additionally, the interview format focused on provider perspectives, lacking patient voice. Incorporating patient voices would be crucial for understanding patient experiences during the perioperative period. Fifth, we recognize that participants might provide responses that are more socially acceptable than their true opinions, a social desirability bias, creating complexities in interpreting findings. Finally, translation of the Amharic interviews may have introduced some cultural ambiguity or loss of context; Amharic, while Ethiopia’s official language in healthcare settings, might not be all participants’ first language, potentially obscuring data subtleties. However, the research team’s composition of primarily perioperative clinicians mitigated this concern. Their insider perspective provided valuable context and facilitated a nuanced interpretation of the participants’ narratives.

## Conclusions

This mixed-methods research reveals contextual challenges in infrastructure, workforce, service delivery, financing, and information management that hinder quality perioperative care in three teaching hospitals in southern Ethiopia. Key recommendations include increasing health financing and insurance coverage for essential surgery, incentivizing workforce expansion and retention, procuring equipment strategically, fostering perioperative safety culture through training and protocol implementation, strengthening digital health systems, and addressing leadership and governance deficiencies. Research on perioperative implementation and sustainability science can strengthen the evidence base for change. Exploring the influence of sociopolitical factors on national surgical system strengthening efforts is also essential. Continuous innovation, regular capacity evaluations, and qualitative insights are crucial for directing evidence-based policy and monitoring for safe and affordable surgery for all.

### Supplementary Information

Additional file1. A joint display table linking the key survey findings of perioperative hospital capacity to quotes of contextual challenges perceived by clinicians.

## Data Availability

The datasets used and/or analyzed during the current study are available from the corresponding author on reasonable request.

## Abbreviations

CE: Catastrophic Expenditure
HCP: Healthcare Professional
HIMS: Health Information Management System
LCoGS: Lancet Commission on Global Surgery
LMICs: Low- or Middle-Income Countries
MMR: Mixed-Methods Research
NSOAP: National Surgical Obstetric and Anesthesia Planning
PGSSC: Program in Global Surgery and Social Change
PSC: Patient Safety Culture
QI: Quality Improvement
SAT: Surgical Assessment Tool
SaLTS: Saving Lives Through Safe Surgery
SOA: Surgery Obstetric and Anesthesia
WHO: World Health Organization
