# Supplementary material for: Perioperative capacity and contextual challenges in teaching hospitals of southern Ethiopia: explanatory sequential mixed-methods research
Source: Perioper Med (Lond). 2024 Jun 22;13:61. doi: 10.1186/s13741-024-00423-6 (PMC11193207; doi:10.1186/s13741-024-00423-6)
Supplement: Supplementary file 1 — Additional file1. A joint display table linking the key survey findings of perioperative hospital capacity to quotes of contextual challenges perceived by clinicians. [file 13741_2024_423_MOESM1_ESM.pdf]

Additional file 1 A joint display table linking the key quantitative and qualitative findings

Domain	Quantitative findings	Linking activity*	Qualitative findings	Categories
**Infrastructure**	The surgical capacity was 115 surgeries per 100,000 people.		The workflow in the operating room is inefficient and the number of OR tables isn’t adequate. (P2)	Inadequate OR tables and inefficient workflow
	Radiology service was available 25-50% of the time.		While there are CT scans, MRI and so on, it is often said that these services have been malfunctioning; patients are subjected to added expenses; this is what we often see. (P16)	Shortage and malfunctioning of equipment
	Blood and anesthetic drugs were available only 25-50% and 50-75% of the time, respectively.		There are more cases of blood shortage related cancellations than for other reasons. (P3)	Shortage of blood
			There has been cancellation of surgical cases due to shortage of anesthetic medications. (P1)	Shortage of drugs and other supplies
**Service delivery**	Management guidelines were rarely used (1-25 % of the time) while the WHO’s surgical safety checklist was used almost always (76-99% of the time).		There is incomplete documentation of perioperative care and we do not always use the WHO safety checklist. (P5)	Poor patient safety culture and noncompliance to management guidelines
			The surgeon may not be aware of the patient's medical history, or the nurse may not be aware of the surgeon's instructions. (P12)	Lack of interprofessional, team-based perioperative care
			Projects often begin, but we don’t see a sustainable change. The baseline problem usually recurs in the middle of the projects or after completion. (P8)	Unsustainable quality improvement projects
**Workforce**	There were 0.58 surgical, obstetrics, and anesthesia specialists per 100,000 people.		There is still a shortage of human power per capacity this hospital can deliver. (P 1)	Shortage of workforce
			The country’s salary for health professionals doesn’t go with standard of living, especially from the current inflation. (P6)	Job dissatisfaction
**Financing**	Less than 10% of patients had perioperative insurance coverage.		There are small number of patients with health insurance; most of the supplies are not found in hospital; So, it means that patients buying from a private pharmacy will be covering the expense by their-selves, and you know it is unintended. (P18)	Inadequate public health insurance coverage
			The biggest problem is that availability of the hospital's supply of anesthetic drugs and other surgical supplies. It runs out quickly and often. There is a need to upscale surgical care financing. (P8)	Inadequate hospital funding
			It is a moral burden as a clinician to decide on such issues [when patients cannot afford surgical care]. I believe hospitals should have a mechanism for such patients. (P19)	Unaffordable perioperative cost and ethical dilemmas
**Information management**	Perioperative outcome data is assessed sometimes (only 26 - 50% of the time).		We need a system in place to collect data and track patient outcomes. We also need funding to support research projects. (P6)	Lack of funding for quality improvement projects
	100% of surveyed hospitals had paper-based perioperative documentation.		Once patients are triaged to each ward, they get a paper-based medical card; some medical charts may be lost; and sometimes we have difficulty obtaining a prior medical history. (9)	Inadequate information management system
**Leadership and Sociopolitical landscape**	..		Domestic civil and political unrest and global market instability have a significant impact on our medical delivery. (P12)	Sociopolitical unrest and poor leadership and governance

* The linking activity searches for related quantitative findings and chooses appropriate qualitative quotes to illustrate them. Meta-inferences between the findings are shown with colored arrows. Red represents convergence, indicating agreement; blue represents complementarity, showing different but non-contradictory interpretations; and black represents expansion, allowing for overlap and further interpretation. There was no divergence (conflicting interpretations) during the linking activity.
